# Supplementary material for: Chromothripsis during telomere crisis is independent of NHEJ, and consistent with a replicative origin
Source: Genome Res. 2019 May;29(5):737–49. doi: 10.1101/gr.240705.118 (PMC6499312; doi:10.1101/gr.240705.118)
Supplement: Supplemental Material [file supp_gr.240705.118_Supplemental_file_1.zip › contigs/annotated_contigs/DB50/contig.2.DB50_length_635_mean_cov_5.1968503937.docx]

**DB50_length_635_mean_cov_5.1968503937**

```
TGATCAATTTTATCAATCAAGCCAGTCTGTGTTACGTACAAAACTACACAATCTATTTACTATTTACTGCGTGCCTCTAGGATTATTCA
 >chr4:19170938-19171168 - E=1e-126
ATATCTGTATAATGTGGCAAGGATGCAGATATAATAGACATGGTCCCCTCCTTGAAGGAAAACATTGCAATGTATCTGAATTATATGTG

TGGACATCAGAGTATCCCTTTCTCAATCACATGTTGATATTCTTTTCTTTTT|ACATTTTTA|CAGAATGTTCAATCTGTACTTTGTGA
                                                              >chr4:19168898-19169294 -
ATGGTGGCTTCTCTTTTGACCCAGGTAATGAGCTGTTAACTCTTTAAGAAGAATACAACACAGCTCCATGGAGGCCTCTCTGGCCTAGG
 E=1e-226
AACAATGCTAGGCATTATGCATACACTATCTCACCTTATCCTTACAACAAATTTACAACATGGGGCCAAGGGACAAAATGAGAAGTGAA

GTGACAAAGTCTAGGTTGCTTCCTGAGGTCACACATAAAATGAAGAGCCAAGCTGGGCTTCAGACTTAGGAATGTCTGCCTTCAAATCA

ACACACCTCCCACAATCTTAAACTCTCTTCTCAGAGTAAGGTTGTTATTTAGATAGATTTTTCATATATTGCATGTTTCATTGTGTTGA

TTAATTCAATAAAA
```
